# Synergetic Effect of Different Carrier Dynamics in Pm6:Y6:ITIC-M Ternary Cascade Energy Level System

**DOI:** 10.3390/polym13152398

**Published:** 2021-07-22

**Authors:** Zicha Li, Dandan Song, Zheng Xu, Bo Qiao, Suling Zhao, S. Wageh, Ahmed A Al-Ghamdi, Xiaomin Huo

**Affiliations:** 1 Key Laboratory of Luminescence and Optical Information, Beijing Jiaotong University, Ministry of Education, Bejjing 100044, China; 16118446@bjtu.edu.cn (Z.L.); ddsong@bjtu.edu.cn (D.S.); zhengxu@bjtu.edu.cn (Z.X.); boqiao@bjtu.edu.cn (B.Q.); 20118041@bjtu.edu.cn (X.H.); 2 Department of Physics, Faculty of Science, King Abdulaziz University, Jeddah 21589, Saudi Arabia; AGAMDI@kau.edu.cn

**Keywords:** ternary solar cells, carrier dynamics, electroluminescence

## Abstract

Although reported ternary polymer solar cells have higher power conversion efficiency than binary polymers, the mechanism of exciton separation and charge transport in this complex ternary system is still unclear. Herein, based on PM6:Y6:ITIC-M ternary solar cells, we combine the technique of luminescence spectroscopy, including electroluminescence (EL) and photoluminescence (PL) with photovoltaic measurements, to understand clearly the detailed roles of ITIC-M as the third component in the contribution of device performance. The results show that ITIC-M can form the alloy-like composite with Y6 but leave individual Y6 acceptor to conduct charge transfer with PM6 donor, which improves *V_oc_* but decreases *J_sc_* because of poor charge transfer capacity of ITIC-M. Meanwhile, the energy transfer from PM6 to ITIC-M exists in the active layers; small IE suppresses exciton dissociation. Deteriorating performance of solar cells demonstrates that, except for complementary absorption spectrum and suitable energy levels in PM6:Y6:ITIC-M system, the synergetic effects of carrier dynamics among different organic materials play an important role in influencing the performance of ternary solar cells.

## 1. Introduction

The development of sustainable energy can help alleviate the energy crisis and environmental problems. In particular, organic solar cells (OSCs), as a kind of strong candidate for the new energy sources, have attracted much attention due to their low cost, light weight, mechanical flexibility, translucency, fast roll-to-roll printing method and non-toxicity [1,2,3,4]. So far, many strategies have been developed to improve the performance of OSCs, such as the synthesis of high-efficiency photoelectric materials [5,6], optimization of blend film morphology [7], tandem cell approach [8] and so on. Therein, ternary organic solar cells (TOSCs) have an active layer composed of three light-collecting materials with a wide complementary range of light absorption similar to tandem cells but with a simple structure design which makes them attract widespread attention [9,10,11]. The working mechanism of TOSCs is summarized as charge transfer, energy transfer or as parallel-connected tandem cells. The charge can be transferred from the original materials to the add-on component or from the add-on component to the original materials or between the original materials [11,12,13]. If the charge transfer network is independently in TOSCs [14], TOSCs work the same as parallel-connected tandem cells. For energy transfer, it includes a long-distance Forster resonance energy transfer (FRET) and a short-distance Dexter energy transfer among three components [15,16]. In recent years, the non-fullerene acceptors (NFA) have been widely used in TOSCs, which exhibit great potential in providing morphological advantages, reducing energy loss and expanding absorption range [17,18]. In particular, it was reported that TOSCs based on NFAs achieved a record power conversion efficiency (PCE) of 17.6% [19]. Peng’s group reported that in PBDB-T: PTB7-Th: SFBRCN ternary solar cells, there exist multiple energy transfer pathways from PBDB-T to PTB7-Th, and from SFBRCN to the above two polymer donors [20]. Chen’s group reported a new non-fullerene acceptor named BTP-M as the third component for the PM6:Y6 binary system, where an alloy-like composite is formed between Y6 and BTP-M [21]. Ge et al. reported a non-fullerene acceptor DTF-IC as the third component in the PBDB-T:IT-M system, which is able to provide a cascading energy level between host donor and acceptor to improve charge transfer [22]. Different carrier dynamics are mentioned in many previous reports. However, the synergetic effect of different carrier dynamics in TOSCs is rarely mentioned but it is very important to affect the efficiency of TOSCs.

In this article, we fabricated various ternary cell devices by incorporating ITIC-M (3,9-bis((Z)-1-(6-(dicyanomethylene)-2-methyl-5,6-dihydro-6H-cyclopenta[b]thiophen-6-one-5-yl)ethylene)-5,5,11,11-tetrakis(4-hexylphenyl)dithieno [2,3-d:2′,3′-d′]-sindaceno [1,2-b:5,6-b′]dithiophene), an NFA as the additional acceptor material into (poly[(2,6-(4,8-bis(5-(2-ethylhexyl-3-fleoro)thiophen-2-yl)-benzo[1,2-b:4,5-b′]dithiophene))-alt-(5,5-(1′,3′-di-2-thienyl-5′,7′-bis(2-ethylhexyl)benzo[1′,2′-c:4′,5′-c′]dithiophene-4,8-dione)]) PM6:Y6 (2,20-((2Z,20Z)-((12,13-bis(2-ethylhexyl)-3,9-diundecyl-12,13-dihydro-[1,2,5]thiadiazolo[3,4-e]thieno[2″,30′:4′,50]thieno[20,30:4,5]pyrrolo[3,2-g]thieno[20,30:4,5]thieno[3,2-b]indole-2,10-diyl)bis(methanylylidene))bis(5,6-difluoro-3-oxo-2,3-dihydro-1H-indene-2,1-diylidene))dimalononitrile) host blend films. The ITIC-M shows an absorption spectrum from 500 nm to 800 nm and forms the complementary absorption spectrum with PM6 and Y6, finally increasing the photon harvesting of solar cells. Besides, the highest occupied molecular orbital (HOMO) energy level and the lowest unoccupied molecular orbital (LUMO) energy level of ITIC-M are both located between that of PM6 and Y6, respectively, and Y6 and ITIC-M form an alloy acceptor to improve the open circuit voltage (*V_oc_*). However, lower short circuit current (*J_sc_*) and fill factor (FF) occur in ternary solar cells, which is contrary to the improved absorption. To account for the mechanism of carrier dynamics in ternary solar cells, EL spectroscopy as a simple measurement was utilized to analyze the carrier recombination by combining with PL spectroscopy. It is demonstrated that electrons will accumulate in the alloy acceptor because of the synergy between inefficient extraction and decreased exciton dissociation in ternary solar cells. Consequently, the increasing recombination leads to a decrease of *J_sc_* and FF.

## 2. Materials and Methods

Materials: PM6, Y6, ITIC-M and PDINO were purchased from Solarmer Energy Inc., China, Beijing and used as received. Chloroform (CF) and 1, 8-diiodooctane (DIO) were commercially procured from Sigma-Aldrich, USA, Shanghai branch with purity greater than 99.9% and 98.0%, respectively.

**Figure 1:**
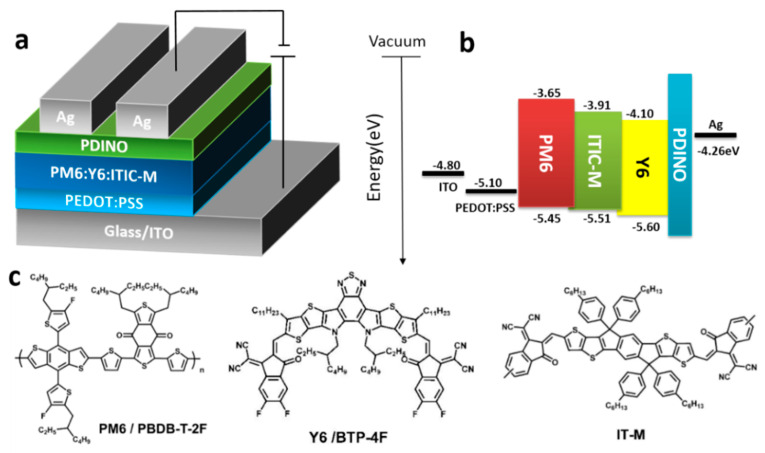
(**a**) The structure of solar cells device, (**b**) the energy level diagram and (**c**) the molecular structure of the polymer donor PM6 and the non-fullerene acceptor Y6 and ITIC-M.

Solution preparation and device fabrication: According to the previous report [23], a 16 mg mL^−1^ PM6:Y6 solution with a weight ratio of 1:1.2 (*w*/*w*) and ternary solutions mixed as blend were prepared in CF and then heated and stirred at 45 °C for 3 h. Next, 0.5 v% DIO as additive was added into dissolved solutions an hour before spin coating the active layer. PDINO was mixed into methyl alcohol with concentration of 1 mg mL^−1^. Ternary solar cells were fabricated in two batches by using the conventional structure of ITO/PEDOT: PSS/active layer film/PDINO/Ag, as shown in Figure 1a. 

As the solutions for ternary devices, in one batch, the polymer donor to the acceptor ratio was kept constant as 1:1.2 and the ratio of Y6 to ITIC-M was changed. In another batch, the ratio of PM6 to Y6 was kept as 1:1.2, and the content of ITIC-M in the bulk-heterojunction (BHJ) was changed. The prepared active layer films and corresponding devices are as follows:

Film 1: PM6: Acceptor (Y6:ITIC-M = 1:0) = 1:1.2
Film 2: PM6: Acceptor (Y6:ITIC-M = 0.9:0.1) = 1:1.2
Film 3: PM6: Acceptor (Y6:ITIC-M = 0.8:0.2) = 1:1.2
Film 4: PM6: Acceptor (Y6:ITIC-M = 0.6:0.4) = 1:1.2
Film 5: PM6: Acceptor (Y6:ITIC-M = 0:1) = 1:1.2
Film 6: PM6:Y6:ITIC-M = 1:1.2:0.1
Film 7: PM6:Y6:ITIC-M = 1:1.2:0.2
Film 8: PM6:Y6:ITIC-M = 1:1.2:0.4
Film 9: PM6:Y6:ITIC-M = 1:1.2:0.6
Film 10: PM6:Y6:ITIC-M = 1:1.2: 1
Device 1–10: ITO/PEDOT: PSS/film1–10/PDINO/Ag

First, indium tin oxide (ITO) coated glass substrates (sheet resistance of 15 ohm/square) were cleaned ultrasonically in a cleaning agent, deionized water and ethanol for 30 min, respectively, and then immediately blow-dried by high-density nitrogen, followed by plasma UV processing for 90 s. A highly conducting polymer PEDOT:PSS was spin coated onto the treated ITO substrates from an aqueous solution at 5000 rpm for 30 s, then these ITO coated with PEDOT:PSS were annealed at 150 °C for 10 min. Next, the solution used as active layer was prepared through spin coating at 3000 rpm for 30 s in N_2_-filling glove box and the prepared activity was annealed at 80 °C for 10 min and thoroughly dried in vacuum chamber for 1 h. After that, PDINO electron transport layer was spin coated on the active layer at 3000 rpm for 50 s. Finally, Ag cathodes (with the film thickness of 1000 Å) were deposited on the top at rate of 1.5 Å/s under a vacuum pressure of 2 × 10^−4^ Pa. The active area of solar cells is 4 mm^2^.

Measurement and characterization: The *J*-*V* characteristic curves of solar cells were measured with a Keithley 2400 source equipment unit under AM 1.5 G simulated solar illumination with an intensity of 100 mW cm^−2^. The EQE measurements were performed using a QE/IPCE Measurements Solar Cell Scan 100 (ZOLIX, Beijing, China) system equipped with calibrated silicon photodiode as the reference cell. PL spectra were acquired using a modified Horiba FL1000. EL spectra were performed by using Keithley 2410 to support bias and detectors in Horiba FL 1000. The UV−vis absorption spectrum was acquired on Shimadzu UV-3101 PC spectrometer. The surface morphology characteristics and 3D images were measured by atomic force measurement (AFM, MFP-3D Infinity).

## 3. Results and Discussion

The device structure and HOMO energy level and LUMO energy level of component materials used in devices are shown in Figure 1a,b, respectively. The molecular structures of the organic polymer donor PM6, non-fullerene acceptor Y6 and ITIC-M used as the photoactive layer are shown in Figure 1c. HOMO energy level (−5.58 eV) and LUMO energy level (−3.91 eV) of ITIC-M is between that of PM6 and Y6, respectively, which will form cascade energy level, as we expected.

**Figure 2:**
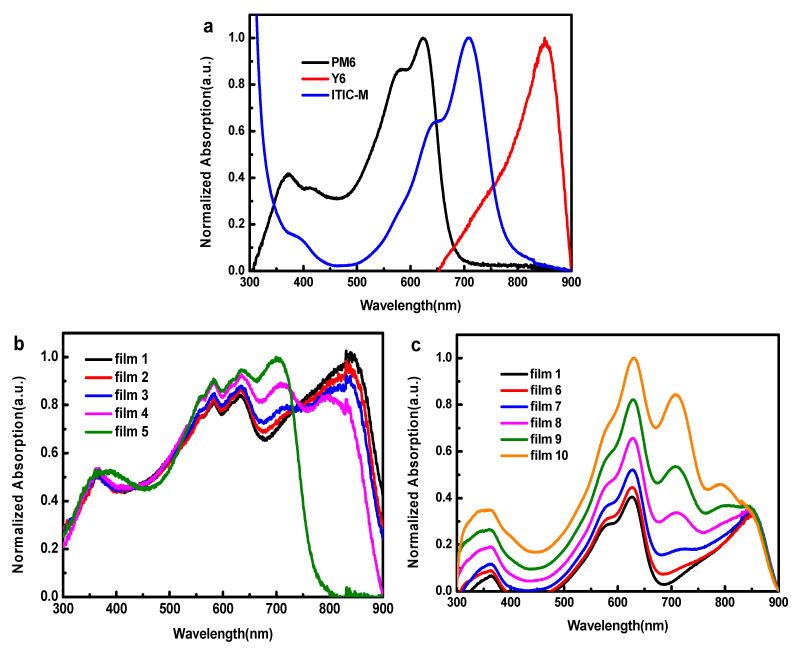
Absorption spectra of (**a**) pure PM6, Y6 and ITIC-M films normalized, respectively, at their peak, (**b**) blend films 1–5 normalized at 450 nm, (**c**) blend films 1, 6–10 normalized at 845 nm.

The absorption spectra of pure films and D:A blend films were measured and the normalized absorption spectra are shown in Figure 2a–c. PM6 film exhibits wide photon harvesting range with a major absorption peak at 630 nm. Y6 and ITIC-M films show strong photon harvesting ability in long wavelength range with the absorption peak at 700 nm and 820 nm, respectively, showing obviously complementary absorption spectra. As shown in Figure 2b,c, incorporation of ITIC-M could help to absorb light between 550 nm and 800 nm.

**Figure 3:**
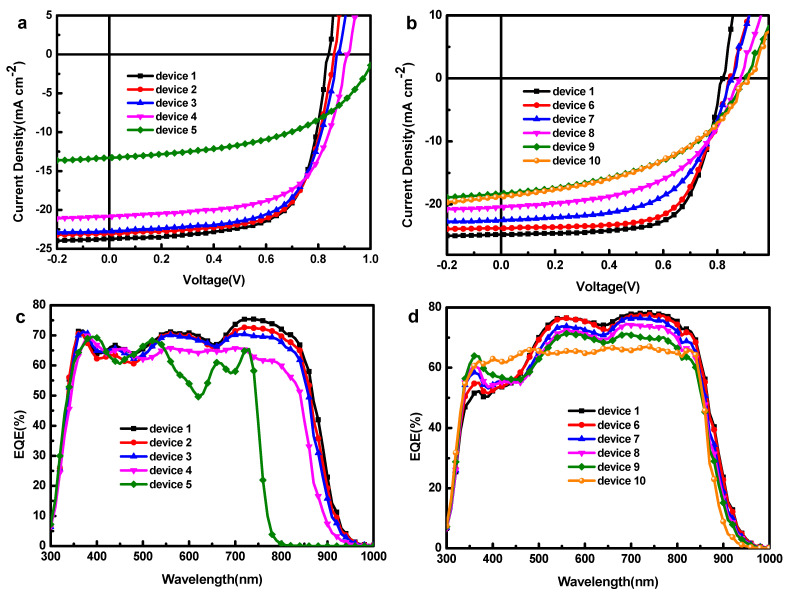
*J*-*V* characteristics of (**a**) devices 1 to 5, (**b**) devices 1, 6 to 10, EQE curves of (**c**) device 1 to 5, (**d**) device 1, 6 to 10. (R_sh_) and series (R_s_) resistances of these devices are from their *J-V* curves.

Figure 3a,b show the current density voltage (*J-V*) characteristics of different devices under AM1.5G illumination with the intensity of 100 mW cm^−2^. Table 1 and Table 2 summarize the OPV parameters of all devices. Device 1 exhibits a PCE of 14.03% with a value of *J_sc_* of 24.80 mA cm^−2^, *V_oc_* of 0.83 V and FF of 68.30%. *V_oc_* increases with the augment ratio of ITIC-M in acceptors from 0.83 V to 0.91 V (device 1 to device 4), and obtains the highest value of 1.02 V in PM6:ITIC-M binary solar cell (device 5). The same *V_oc_* increase (from 0.85 V to 0.91 V) can be observed in devices 6 to device 10, which is attributable to the alloy acceptor formed by Y6 and ITIC-M as shown in later discussion. However, *J_sc_* and FF decrease sharply with addition of ITIC-M and finally the value of PCE decreases. We determined the shunt (R_sh_) and series (R_s_) resistances of these devices from their *J*-*V* curves (Table 1) to evaluate their bulk and interfacial resistances (R_s_), as well as the leakage current and free carrier recombination in their BHJ (R_sh_) of the devices. The values of R_s_ in devices incorporating ITIC-M are higher than PM6:Y6 binary device (device 1), implying more defects at the interfaces and within the BHJ blend films of these devices. Moreover, lower values of R_sh_ suggest higher degrees of free carrier recombination. 

As external quantum efficiency (EQE) results show in Figure 3c,d, the EQE of device 2 and 3 is lower than that of device 1 from 665 nm to 900 nm and the EQE from 300 nm to 650 nm has no significant change. This means that photogenerated carriers corresponding to the absorption of PM6 are collected similarly in three devices. However, as the ratio of ITIC-M increases, the carriers generated by ITIC-M and PM6 decrease, even as their light absorption increases. This may be due to the fact that excitons formed in ITIC-M and PM6 cannot be dissociated at their interface or ITIC-M cannot transfer electrons effectively. In device 4, the EQE from 475 nm to 750 nm has an obvious drop while the EQE of device 5 declines more drastically. In devices 6, 7, 8 and 9, the EQE from 450 nm to 900 nm drops with increase of ITIC-M, but rises a little from 350 nm to 450 nm, which is consistent with the changing of the absorption spectrum. Besides, the EQE of device 10 from 500 nm to 900 nm decreases dramatically. These drops of EQE are contrary to the enhancement of light absorption (Figure 3). It can be speculated that the addition of ITIC-M suppresses the exciton generated by absorbing light between 525 nm and 850 nm to dissociate into free carriers or suppresses these free carriers to transfer to electrode.

**Figure 4:**
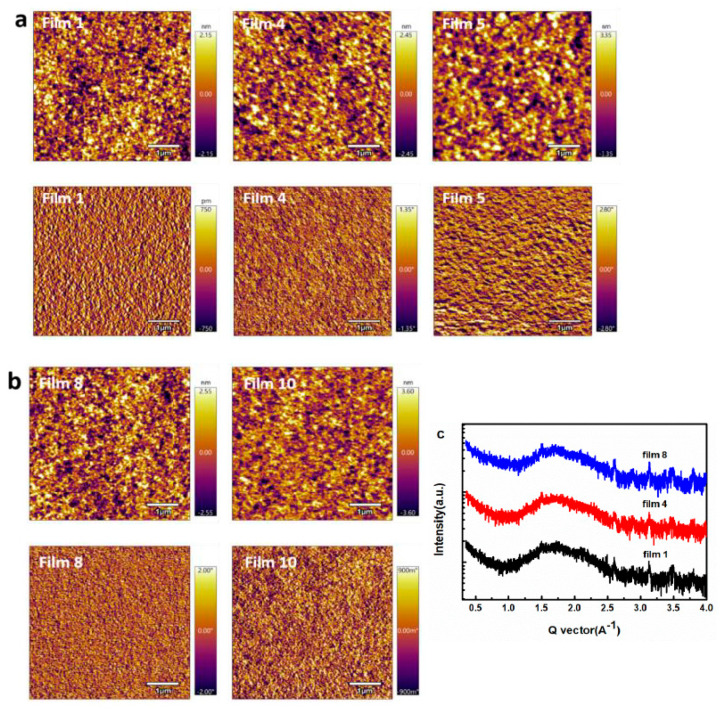
AFM topography images (above) and the corresponding phase images (below) of 5 × 5 μm^2^ area of (**a**) film 1, film 4, film 5, (**b**) film 8 and film 10, (**c**) GIXRD with out-of-plane scattering geometry for film 1, 4, 8.

The microscopic morphology of the active layer plays a decisive role in the generation and transmission of charges in devices [24,25]. In order to understand the physical mechanism of performance decrease, firstly, we investigated the effects of the third content ITIC-M on the morphology of the PM6:Y6 blend film. The surface and bulk morphologies of five blend films (film 1, 4, 5, 8, 10) as examples were characterized by tapping-mode atomic force microscopy (AFM), as shown in Figure 4. The root mean square roughness (RMS) of different blend films are 1.078 nm (film 1), 1.217 nm (film 4), 1.652 nm (film 5), 1.270 nm (film 8) and 1.507 nm (film 10). RMS values reveal that the surface of blend films become a little rougher by adding ITIC-M, which is plausible as larger phase segregation emerges by incorporating ITIC-M as well as can be observed in the phase separation of blend films from the phase images in Figure 4. The rather rough surface increases the interface resistance, which is not conductive to the charge transport and extraction, and the larger phase separation due to adding ITIC-M will reduce the exciton dissociation interface. To further investigate the effect of ITIC-M on the blend film, one-dimensional (1D) grazing incidence X-ray diffraction (GIXRD) was used in film 1, 4 and 8. As shown in Figure 4c, three films exhibit a weak diffraction peak at 1.72 Å^−1^ in the out-of-plane direction, which corresponds to that of PM6. The almost identical locating at the same position and same intensity of GIXRD peak indicates that ITIC-M generates nearly no difference in polymer (PM6) crystallinity.

**Figure 5:**
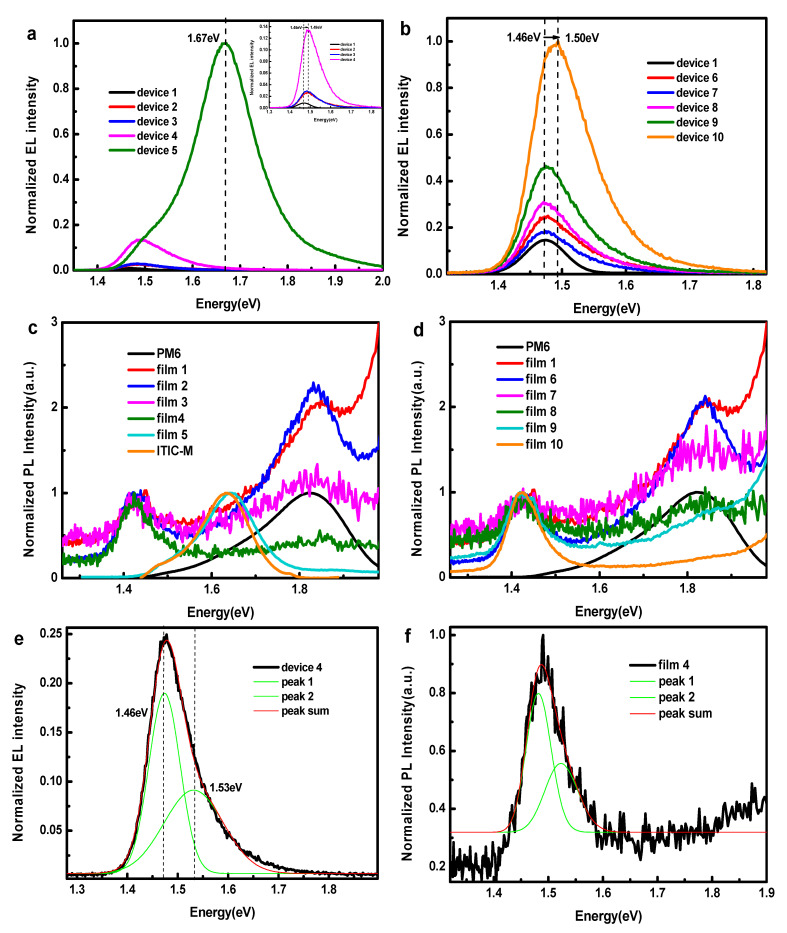
EL spectroscopy of (**a**) devices 1 to 5 (inset image is EL spectroscopy of film 1 to 4), (**b**) devices 1, 6 to 10, PL spectra of (**c**) pure PM6 film, ITIC-M film and films 1 to 5, (**d**) pure PM6 film and films 1, 6 to 10, excited at 550 nm. Multiple peaks, Gaussian fitting of (**e**) EL spectroscopy of device 4 and (**f**) PL spectroscopy of film 4.

According to the above results, the carrier dynamic inside devices has changed after the addition of ITIC-M and the new carrier dynamic leads to a decreasing performance. To further understand the charge separation and charge transport process in ternary devices, we used EL spectrometry to study the exciton decay way and the charge transfer states (CTSs) of devices. The EL spectra of devices with different content of ITIC-M are shown in Figure 5a,b. The emission peak positions of CT states (E_CT_) vary in different devices; the E_CT_ [26,27] becomes higher from 1.46 eV of device 1 to 1.49 eV of device 4, then locates at 1.67 eV in device 5 with the active layer PM6:ITIC-M. For device 6 to device 10 compared with device 1, E_CT_ also changes from 1.46 eV to 1.50 eV with the increase of ITIC-M content. The changes of E_CT_ indicate that it is plausible to consider Y6 and ITIC-M forming an alloy acceptor, and the E_CT_ reaches maximum (1.67 eV) in PM6:ITIC-M binary device, which is consistent with the variation of *V_oc_*.

PL spectra were measured to further analyze the energy transfer mechanism in PM6:Y6:ITIC-M blend film excited by 550 nm as shown in Figure 5c,d. In the blend film of PM6:Y6 system, there are two peaks: 1.83 eV (678 nm) is from pure PM6 and 1.47 eV (846 nm) is from the recombination of CT states corresponding to the HOMO of PM6 and LUMO of Y6. Furthermore, in the blend film of PM6: ITIC-M, only one peak locates at 1.66 eV (746 nm), which corresponds to the recombination of CT states between HOMO of PM6 and LUMO of ITIC-M, and ITIC-M can almost quench PL of PM6. Although the large overlap of PM6 PL (678 nm) and ITIC-M absorption (700 nm) triggers efficient energy transfer from PM6 to Y6, the almost identical PL of CT state of PM6:ITIC-M with PL of ITIC-M means the HOMO of PM6 closes that of ITIC-M, which determines that the ionization energy (IE) is not enough to dissociate excitons in ITIC-M into free carriers by transferring holes from ITIC-M to PM6 efficiently [28]. The multiple peaks Gaussian fitting results in Figure 5e reveal that there are two peaks in EL spectroscopy of ternary solar cells, one is at 1.46 eV and another is at 1.53 eV, and the multiple peaks Gaussian fitting results of PL spectroscopy in Figure 5f are consistent with those of EL. Coexistence of EL peaks at 1.46 eV and 1.53 eV means that excitons can be dissociated in the interface of PM6:Y6 and between PM6 and Y6-ITIC-M alloy acceptor while the content of Y6 is larger than ITIC-M. However, in the PL spectra of ternary films, 1.67 eV (746 nm) corresponding to the CT emission of PM6:ITIC-M was missing, and the PL peak of PM6 decreased but the peak of CT states between PM6 and Y6 as well as between PM6 and Y6–ITIC-M alloy (shown in Figure 5e,f) increased along with the increasing content of ITIC-M. This means that increasing Y6- ITIC-M alloy is formed and helps to decrease excitons of PM6, but more carrier recombination [29] is the result at the interface of PM6 and Y6-ITIC-M alloy, which is one reason to cause low *J_sc_* and FF. Therefore, it is concluded that by adding ITIC-M, even the exciton of PM6 is dissociated easily, but the recombination probability of CT states of PM6:Y6:ITIC-M increased, which may be due to the deterioration of charge transportation and increase of defect density.

**Figure 6:**
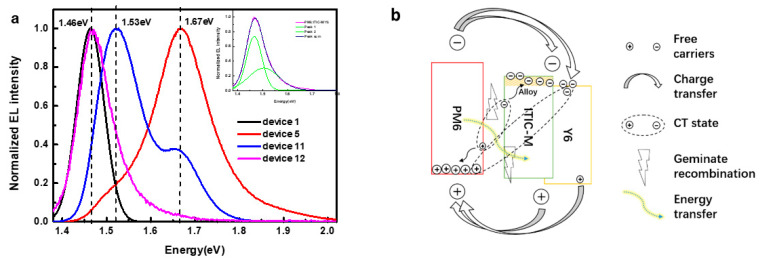
(**a**) EL spectroscopy of device 1, 5, 11 and 12 (inset image is multiple peaks Gaussian fitting of device 12), (**b**) charge transfer model of PM6:Y6:ITIC-M ternary solar cells.

To obtain more information of exciton separation and charge transfer mechanism in ternary films, we measured the EL spectra of devices by using the structure of ITO/PEDOT:PSS/PM6:Y6/ITIC-M/PDINO/Ag and ITO/PEDOT:PSS/PM6:ITIC-M/Y6/PDINO/Ag, named as device 11 and device 12, respectively, as shown in Figure 6a. The EL peak position of device 12 moves a little to the high-energy side compared with device 1, and the E_CT_ emission of PM6:ITIC-M almost disappears. According to the result of multiple peaks Gaussian fitting (Figure 6a, inset), all ITIC-M form an alloy acceptor with Y6 and extra PM6:Y6 conduct charge transfer at their interface. In device 11, disappearance of EL peak located at 1.46 eV and appearance of the EL peaks located at 1.53 eV and at 1.67 eV indicate that carriers are recombined at the interface between PM6 and Y6:ITIC-M alloy acceptor and between PM6 and ITIC-M, which proves the bad electron transportation in ITIC-M. Combining EL results of device 11 with the other devices, it can be concluded that parts of ITIC-M and Y6 form an alloy acceptor and extra Y6 or ITIC-M could conduct charge transfer with PM6 alone.

**Figure 7:**
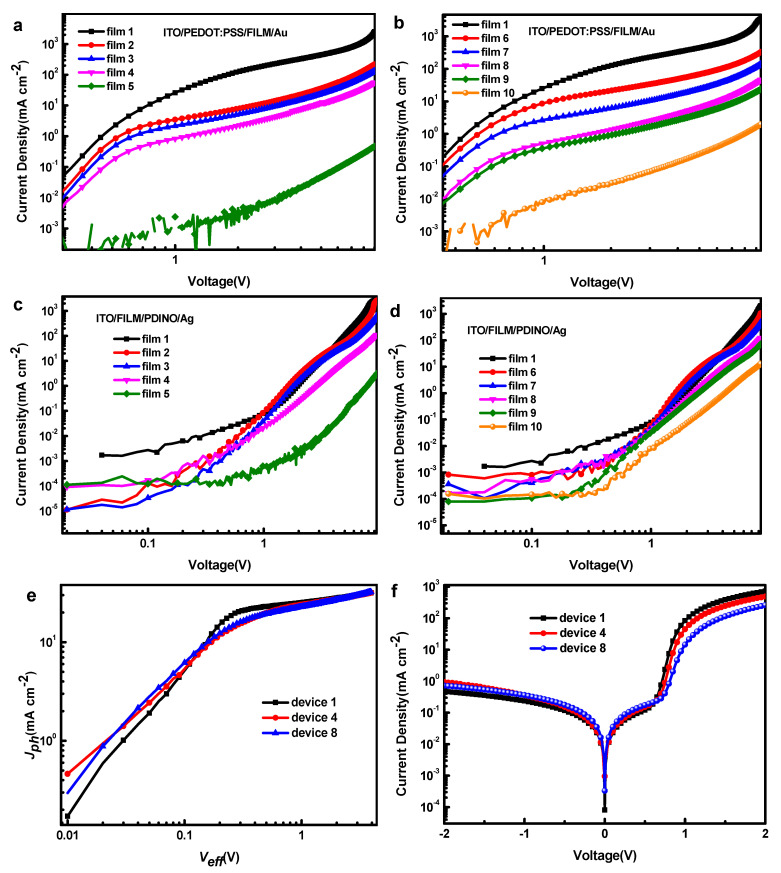
Dark *J*-*V* characteristics of hoe-only devices (**a**) 1 to 5, (**b**) 1, 6 to 10. Dark *J*-*V* characteristics of electron-only devices (**c**) 1 to 5, (**d**) 1, 6 to 10, (**e**) photocurrent density (*J_ph_*) versus effective voltage (*V_eff_*) for devices 1, 4 and 8, (**f**) dark *J-V* curves of devices 1, 4 and 8.

In order to figure out the reason for recombination enhancement, the dark *J-V* characteristics of hole-only and electron-only devices were measured with the structure of ITO/PEDOT: PSS/active layer/Au and ITO/active layer/PDINO/Ag shown in Figure 7. The apparent charge carrier mobility of blend films is evaluated through the spare charge limit current (SCLC) method [30,31]. According to the Mott–Gurney law, the current density is given by (Equation (1)):

(1) $$J=9\varepsilon_{0}\varepsilon_{r}\mu V^{2}/8L^{3}$$

where *J* is the current density, *ε*_0_ is the permittivity of free space, *ε_r_* is the relative dielectric constant of the BHJ layer, *μ* is the charge carrier mobility, *L* is the thickness of the BHJ layer and *V* is the voltage drop across the device [32]. The computed results are shown in Table 3.

The hole mobility and the electron mobility of ternary devices incorporated with ITIC-M all decrease dramatically. In addition, the unbalance between hole and electron mobility would lead to the accumulation of carriers with the lower mobility in the device. This will result in an additional electric field which would hinder the extraction of carriers and increase the radiative recombination [33,34]. Then, the hole and electron trapping states of devices 1, 4 and 8 were calculated from *J*-*V* curves [35]. When the voltage goes beyond the kink point, it means that the trapping states are completely filled, and the trapping state density can be calculated by Equation (2):

(2) $$V_{TFL}=\frac{en_{t}L^{2}}{2\varepsilon_{0}\varepsilon }$$

where *V_TFL_* is the trap filled limit voltage, e is the elementary charge, *L* is the thickness of the active layer, *ε* is the relative dielectric constant of polymer (*ε* = 3), *ε*_0_ is the vacuum permittivity and *n_t_* is the trapping state density.

The electron and hole trapping states of devices 1, 4 and 8 are acquired as 1.77 × 10^17^ cm^−3^, 1.08 × 10^17^ cm^−3^ and 4.84 × 10^16^ cm^−3^ and 2.36 × 10^16^ cm^−3^, 4.13 × 10^16^ cm^−3^ and 2.07 × 10^16^ cm^−3^, respectively. The variation of hole trapping states is coherent with that of hole current that changes a little with the increasing of ITIC-M content. However, even the electron trapping state density decreases with the increasing of ITIC-M content in the active layer, but the electron current decreases dramatically. This means that the electron transport is going to be difficult in the active layer due to the adding of ITIC-M, which is proven by the lower electron mobility in ternary devices with increasing of ITIC-M. It also corresponds to the electroluminescence results detected in PM6:Y6:ITIC-M active layer.

Otherwise, we measured the photocurrent density (*J_ph_*) versus the effective voltage (*V_eff_*) curves for the devices 1, 4 and 8, plotted in Figure 7e. In principle, *J_ph_* is calculated according to [36,37]:

(3) $$J_{ph}=J_{L}-J_{D}$$

where *J_L_* and *J_D_* represent current density under AM 1.5G illumination and in the dark, respectively. *V_eff_* is calculated according to [36,37]:

(4) $$V_{eff}=V_{0}-V_{a}$$

where *V*_0_ stands for the voltage at which *J_ph_* = 0 and *V_a_* is the applied voltage.

We can find that the *J_ph_* of three devices shows linear dependence on the voltage at a low *V_eff_* (<0.1 V), and rapidly reaches saturation at the high *V_eff_* (>1 V). It clearly shows that the devices 4 and 8 have a lower saturation photocurrent density (*J_sat_*) than that of device 1. The charge dissociation probability P (E, T) of both devices determined by *J_ph_*/*J_sat_* under short circuit condition is 89.11%, 84.27% and 84.69%, respectively. Combining P (E, T) and dark *J*-*V* curves (shown in Figure 7f), it is explicit that in PM6:Y6:ITIC-M ternary solar cells, ITIC-M suppresses exciton dissociation and increases recombination with decreasing of *J_sc_* and FF.

## 4. Conclusions

In summary, we utilized ITIC-M as third content incorporated into PM6:Y6 to form ternary solar cells. Combining two simple measurement methods, EL spectroscopy and PL spectroscopy, it is demonstrated that there are different transfer mechanisms in PM6:Y6:ITIC-M ternary solar cells: (i) coexistence of EL peaks at 1.46 eV and 1.53 eV means that parts of ITIC-M and Y6 formed alloy acceptor while individual Y6 exists and conducts charge transfer with PM6 alone, (ii) large overlap of PM6 PL (678 nm) and ITIC-M absorption (700 nm) indicates existence of energy transfer from PM6 to ITIC-M. Small IE is adverse to exciton dissociation and unbalance of carrier mobility leads to accumulation of electrons, finally improving recombination. Except complementary absorption spectrum and suitable energy levels in PM6:Y6:ITIC-M system, carrier dynamics between organic materials play an important role in the performance of ternary solar cells.

## Figures and Tables

**Table 1:** Performance parameters of devices 1 to 5.

Device	*V_oc_* (V)	*J_sc_* (mA cm^−2^)	FF (%)	PCE (%)	R_s_ (Ω cm^2^)	R_sh_ (Ω cm^2^)
1	0.83	24.80	68.30	14.03	4.62	964.16
2	0.86	22.99	67.34	13.34	5.01	812.24
3	0.88	22.68	65.21	12.96	5.73	724.28
4	0.91	20.78	64.13	12.16	6.18	644.32
5	1.02	13.27	51.26	6.96	15.95	571.16

**Table 2:** Performance parameters of devices 1, 6 to 10.

Device	*V_oc_* (V)	*J_sc_* (mA cm^−2^)	FF (%)	PCE (%)	R_s_ (Ω cm^2^)	R_sh_ (Ω cm^2^)
1	0.83	24.80	68.30	14.03	4.62	964.16
6	0.85	23.76	65.86	13.27	6.91	1332.92
7	0.86	22.48	58.79	11.40	8.25	619.36
8	0.89	20.36	53.88	9.77	8.54	421.92
9	0.91	18.32	47.39	7.93	13.04	330.72
10	0.91	18.74	45.62	7.79	13.21	217.64

**Table 3:** Summary of charge carrier mobility calculated by SCLC.

Devices	*μ_h_* (cm^2^ V^−1^ s^−1^)	*μ_e_* (cm^2^ V^−1^ s^−1^)	*μ_h_*/*μ_e_*
1	9.62 × 10^−4^	8.98 × 10^−4^	1.07
2	4.50 × 10^−4^	2.01 × 10^−4^	2.38
3	3.57 × 10^−4^	1.82 × 10^−4^	1.96
4	1.27 × 10^−4^	1.08 × 10^−4^	1.17
5	8.38 × 10^−6^	1.42 × 10^−5^	0.59
6	9.56 × 10^−4^	4.10 × 10^−4^	2.33
7	9.35 × 10^−4^	3.71 × 10^−4^	2.52
8	9.18 × 10^−4^	3.17 × 10^−4^	2.89
9	7.21 × 10^−4^	2.80 × 10^−4^	2.58
10	3.76 × 10^−4^	9.67 × 10^−5^	3.88
